# Hybrid Actuation MEMS Micromirror with Decoupled Piezoelectric Fast Axis and Electromagnetic Slow Axis for Crosstalk Suppression

**DOI:** 10.3390/mi16091072

**Published:** 2025-09-22

**Authors:** Haoxiang Li, Jiapeng Hou, Zheng Gong, Huijun Yu, Yue Liu, Wenjiang Shen

**Affiliations:** 1 School of Nano-Tech and Nano-Bionics, University of Science and Technology of China, Hefei 230026, China; hxli2022@sinano.ac.cn (H.L.); jphou2023@sinano.ac.cn (J.H.); 2 Key Laboratory of Semiconductor Display Materials and Chips, Suzhou Institute of Nano-Tech and Nano-Bionics, Chinese Academy of Sciences, Suzhou 215123, China; 3 State Key Lab of Metal Matrix Composites, School of Materials Science and Engineering, Shanghai Jiao Tong University, Shanghai 200240, China; zgong516@sjtu.edu.cn

**Keywords:** hybrid actuation, piezoelectric actuator, PZT (lead zirconate titanate), LiDAR

## Abstract

Electromagnetic micro-electro-mechanical system (MEMS) micromirrors are widely used in optical scanning systems but often encounter mechanical crosstalk due to the use of shared drive coils. This phenomenon leads to parasitic motion along the slow axis during fast-axis operation, resulting in undesirable elliptical scanning patterns that degrade image quality. To tackle this issue, a hybrid actuation scheme is proposed in which a piezoelectric actuator drives the fast axis through an S-shaped spring structure, achieving a resonance frequency of 792 Hz, while the slow axis is independently driven by an electromagnetic actuator operating in quasi-static mode. Finite element simulations and experimental measurements validate that the proposed decoupled design significantly suppresses mechanical crosstalk. When the fast axis is driven to a 40° optical scan angle, the hybrid system reduces the parasitic slow-axis deflection (typically around 1.43°) to a negligible level, thereby producing a clean single-line scan. The piezoelectric fast axis exhibits a quality factor of Q = 110, while the electromagnetic slow axis achieves a linear 20° deflection at 20 Hz. This hybrid design facilitates a distortion-free field of view measuring 40° × 20° with uniform line spacing, presenting a straightforward and effective solution for high-precision scanning applications such as LiDAR (Light Detection and Ranging) and structured light projection.

## 1. Introduction

MEMS micromirrors have been widely adopted in fields such as LiDAR [1], laser display [2], biomedical imaging [3], and optical communications [2] due to their advantages of small size, low power consumption, and fast response speed. To achieve two-dimensional (2D) beam steering, micromirrors are typically designed with two orthogonal axes to support raster scanning [3] or Lissajous patterns [3].

Various drive mechanisms have been developed for MEMS, each with unique advantages and trade-offs [4]. Piezoelectric actuators [5] are suitable for high-frequency operations [6], offering fast response and low power consumption, but are often limited by nonlinear hysteresis [7] and limited static displacement. Electrostatic actuators [8] are CMOS compatible but require high voltages to generate sufficient torque and are susceptible to pull-in instability. Electromagnetic actuators can achieve large optical scanning angles at relatively low drive currents and exhibit good linearity [9]; however, they rely on permanent magnets and complex packaging. Electrothermal actuators [10] generate significant quasi-static deflection at low voltages, which is advantageous for compact, low-speed devices, but their slow thermal response limits dynamic performance and causes thermal drift during continuous operation.

To overcome the limitations of a single drive method in 2D scanning, researchers have proposed various hybrid or improved drive strategies. Li et al. [11] designed a hybrid structure combining an electrostatic comb and an electrothermal actuator, where the electrostatic drive is responsible for high-speed *x*-axis scanning, while the electrothermal drive provides large-angle, low-frequency deflection in the *y*-axis. This symmetrical design not only combines the fast response and low power consumption of electrostatic drive with the large displacement of electrothermal drive but also effectively reduces crosstalk, making it suitable for scenarios such as projection display and optical imaging. Koh et al. [12] proposed a combination of electrothermal and electromagnetic actuation using electrothermal drive to achieve large-angle deflection in the slow axis and electromagnetic drive to achieve high-speed scanning in the fast axis. This approach enables CMOS-compatible 2D displays at low voltages, suitable for mobile applications. Eun et al. [13] further developed a dual-mode synergistic electrothermal and electromagnetic scheme, achieving low-voltage, large-angle deflection through electrothermal buckling in static mode and high-speed scanning exceeding 10 kHz through electromagnetic drive in resonant mode. This balanced structural symmetry with high rigidity significantly improves the device’s mechanical robustness. Alneamy et al. [14] proposed a dual-drive architecture that combines electromagnetic and electrostatic drives, utilizing electromagnetic drive to achieve wide-angle, high-linearity torsional scanning. Meanwhile, the electrostatic drive simultaneously generates mirror piston motion for focus adjustment, thereby enabling flexible control over both angle and focal length. In addition to hybrid drive, Hwang et al. [15] addressed the processing and integration challenges of piezoelectric micromirrors by proposing a novel drive method using a single commercial bulk PZT plate. This approach maintains a simple structure while providing high driving force and wide-angle scanning capabilities. The integrated electromagnetic sensor also lays the foundation for closed-loop control.

Gu-Stoppel. et al. [16] designed two gimbal-less micromirror structures. In Design A, the torsional axis was positioned between the spring axes, resulting in an asymmetric configuration. This asymmetry caused mode coupling—excitation of one torsional mode inadvertently affected the other, leading to a deviation from the intended linear scan trajectory and producing an elliptical pattern instead. In contrast, Design B achieved a vertical linear scan of 3.4° with a driving voltage as low as 4 V, demonstrating improved decoupling performance. However, due to the relatively small scanning angle, potential inter-axis coupling effects may not have been fully manifested in the experiment. Liu et al. [17] noted that gimbal-less mirrors have high resonance frequencies and high fill factors, enabling fast scanning and high optical efficiency. However, these advantages come at the cost of mechanical coupling between the axes, which reduces scanning accuracy.

In a single-coil electromagnetic micromirror, there is also a coupling problem, which manifests as an “elliptical line” in the scanning pattern. To mitigate this issue, Park et al. [18] designed electrically isolated current paths based on a dual radial magnetic field, enabling independent drive of the fast and slow axes. This solution reduced the slow-axis cross-coupling rate from 2.99% under a single radial magnetic field to 0.45% in actual measurements. Additionally, Park [19] developed a dual-axis electromagnetic micro-scanner without a universal joint torsion mechanism, significantly expanding the scanning angle—the slow-axis scanning angle increased by 1.55 times, and the fast-axis scanning angle increased by 1.97 times. By introducing unique current paths and magnetic circuit components, drive efficiency was further enhanced, and coupling effects were suppressed. Furthermore, Fang et al. [20] proposed a dual-drive coil mirror with a dedicated magnetic circuit composed of four permanent magnets, enabling fully independent control of both axes and effectively eliminating mechanical cross-coupling.

However, these designs often increase manufacturing complexity or package volume, while metal traces on fast axes can cause rapid device fatigue. In this study, we propose a novel hybrid drive strategy, where the fast axis is driven by a piezoelectric actuator, while the slow axis retains the electromagnetic drive. Through simulation and experimental verification, we demonstrate that the proposed mirror significantly reduces parasitic vibrations of the slow axis during fast-axis operation. Time-domain responses indicate that the peak optical offset caused by slow-axis deflection in traditional designs is 1.43°, whereas our design effectively suppresses this phenomenon. As a result, the projection scan line becomes single and sharp, offering a practical and effective alternative solution.

## 2. Principle and Design

### 2.1. Electromagnetic Micromirror

**Figure 1:**
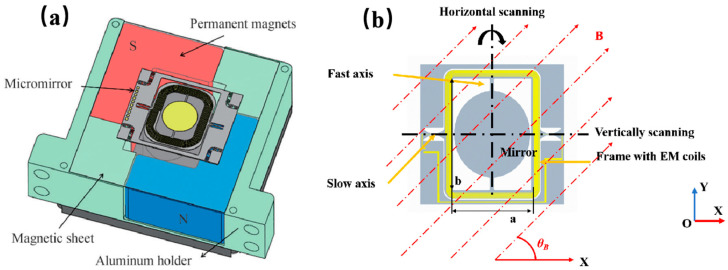
Schematic diagram of a 2D EM MEMS mirror [21]: (**a**) packaging structure; (**b**) drive principle.

As demonstrated in Figure 1, a typical configuration of a two-dimensional (2D) electromagnetic (EM) MEMS micromirror incorporates a pair of L-shaped permanent magnets positioned diagonally around the chip. Orange arrows are used to indicate specific structures, and red arrows represent the magnetic field distribution. This configuration enables a shared set of driving coils (highlighted in yellow) to simultaneously experience magnetic field components along both the X and Y axes. The simultaneous actuation of the micromirror along its two orthogonal axes can be achieved by inputting two sine currents of different frequencies. The low-frequency signal is known to excite the quasi-static slow-axis motion, while the high-frequency signal drives the fast-axis resonant motion. This method generates 2D raster scanning using a single coil structure.

When the fast axis is driven solely by high-frequency sine current *i*(*t*),

(1) $$i(t)={\;I}_{0}\cdot \sin(\omega ft),$$

where *I*_0_ is the peak current, and *ω_f_* is the resonant frequency of the fast axis; the resulting Lorentz torque acting on the mirror can be approximated as

(2) $$T_{f}=i(t)\cdot B\cdot \cos\theta_{B}\sum_{m=0}^{N-1}\;(b+2m\cdot \Delta l),$$

where *B* is the external magnetic field, *N* is the number of coil turns, and *a* and *b* denote the side lengths of the innermost coil turn along the short and long sides of the rectangular frame, respectively. This expression provides an estimate of the total torque contribution from all coil loops, assuming a uniform increment Δ*l* between turns.

Although the fast axis is the primary actuation target, the coil segments (parallel to the *X*-axis) located in the upper and lower regions of the structure also interact with the *Y*-axis component of the magnetic field. These segments are subject to parasitic Lorentz forces, which exert a secondary torque on the slow axis even in the absence of an intentional input signal. The induced force can be described by

(3) $$T_{s}=i(t)\cdot B\cdot \sin\theta_{B}\sum_{m=0}^{N-1}\;(a+2m\cdot \Delta l),$$

The slow axis responds to this unintended excitation like a second-order mass-spring-damper system. Its out-of-band frequency response can be described as

(4) $$\theta_{s}=\frac{T_{s}}{k_{s}}\cdot \frac{1}{\sqrt{[1\;-\;(\frac{W_{f}}{W_{s}}{)}^{2}]+(2\zeta \frac{W_{f}}{W_{s}}{)}^{2}}},$$

where *ω_s_* is the resonant frequency of the slow axis, and *ζ* is the damping ratio. Although the excitation frequency *ω_f_* (equivalent to the fast-axis resonant frequency) is far from the *ω_s_*, the slow axis still produces a small but non-negligible forced response.

**Figure 2:**
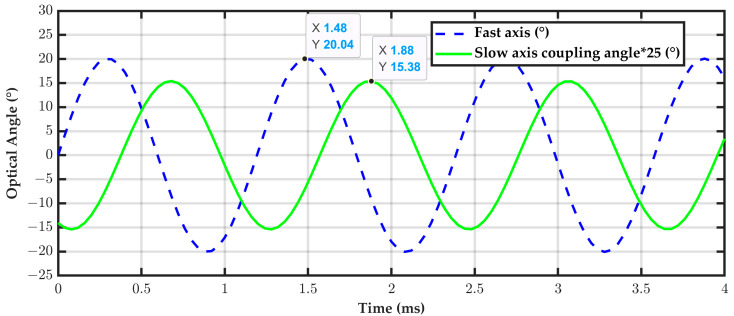
Time-domain analysis of fast axis and coupled slow-axis angles in EM mirror.

In finite element analysis (FEA), a frequency-domain analysis was first performed to evaluate the dynamic response of the fast axis and slow axis. The results were then converted to the time domain. As shown in Figure 2, when the fast axis is driven by a sine wave corresponding to its resonance frequency, the slow axis produces a delayed response with a phase lag of 90°.

Mechanical coupling generates residual oscillations in the slow-axis direction, leading to unintended optical deflection. The peak slow-axis coupling angle is approximately ±0.615°. For phase comparison purposes, the slow-axis coupling angle has been amplified 25 times, as shown as ±15.38 in Figure 2, while the fast-axis deflection angle is ±20°. The scan line will appear in the output in a discrete form, commonly referred to as an “elliptical line”. Such parasitic motion has been proven to adversely affect scanning accuracy, indicating an inherent crosstalk problem in single-coil electromagnetic mirror designs.

### 2.2. Hybrid-Driven Micromirror

**Figure 3:**
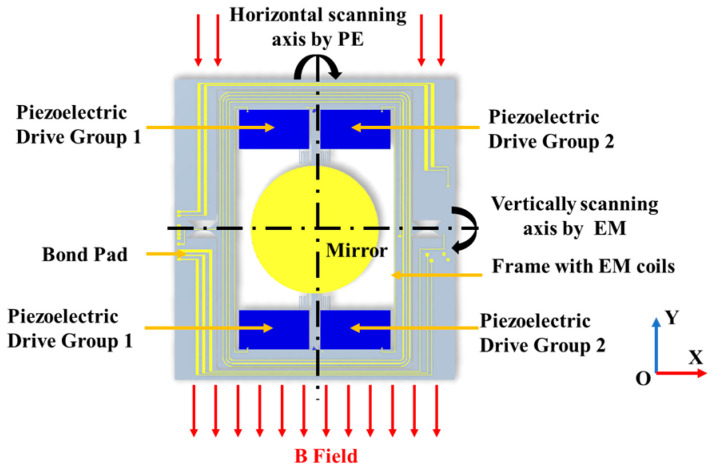
The schematic diagram of the designed 2D MEMS scanning mirror shows that the vertical and horizontal scanning movements are driven by piezoelectric and EM actuation mechanisms, respectively.

Figure 3 presents a schematic illustration of the proposed mirror, which incorporates a hybrid actuation mechanism. The horizontal scanning along the *Y*-axis is facilitated by two groups of piezoelectric actuators. The mechanical energy thus generated is transferred to the mirror plate via an S-shaped spring. When current is applied through the coil on the frame in a magnetic field, mechanical torque is induced, enabling slow scanning along the *X*-axis. Detailed device parameters and piezoelectric coefficients are given in Table 1. The working principles and design considerations of piezoelectric and electromagnetic drive mechanisms are explained in detail below.

The packaging consists of the MEMS chip, CNC packaging holder, two permanent magnets, and an FPC. During assembly, the MEMS chip is first fixed onto the CNC holder using Loctite 380 adhesive, with supports applied around the perimeter to match the boundary conditions used in the simulation. Next, the permanent magnets, selected as N35, are installed into the slots on the back. The FPC is positioned near the MEMS chip and electrically connected through wire bonding at the pads.

**Figure 4:**
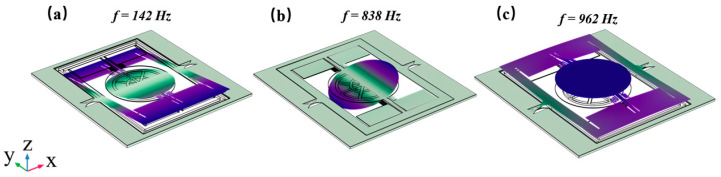
Modal results of the design: (**a**) slow-axis torsion mode; (**b**) fast-axis torsion mode; (**c**) piston mode.

The simulation applies fixed constraints only to the handle Si at the chip periphery to simulate the boundary conditions formed by the adhesive and support structure in the actual package. To balance computational accuracy and efficiency, the model employs a streamlined meshing strategy: hexahedral elements are predominantly generated through sweeping across the entire structure, with regular mapping distributions applied to planar boundaries. Within the PZT layer, thickness-direction meshing is refined into three sub-layers to ensure accurate capture of piezoelectric stress fields, while the silicon substrate, significantly thicker than the PZT, is meshed using a relatively coarse sweeping grid. As shown in Figure 4, the FEA modal frequency analysis indicates that the first mode (142 Hz) of the designed micromirror device exhibits torsional vibration of the mirror frame around the slow axis; the second mode (838 Hz) exhibits resonant torsional vibration of the mirror around the fast axis; and the third mode (962 Hz) describes the mirror piston mode along the *Z*-axis, with a frequency significantly higher than the operational mode.

#### 2.2.1. Piezoelectric Drive

**Figure 5:**
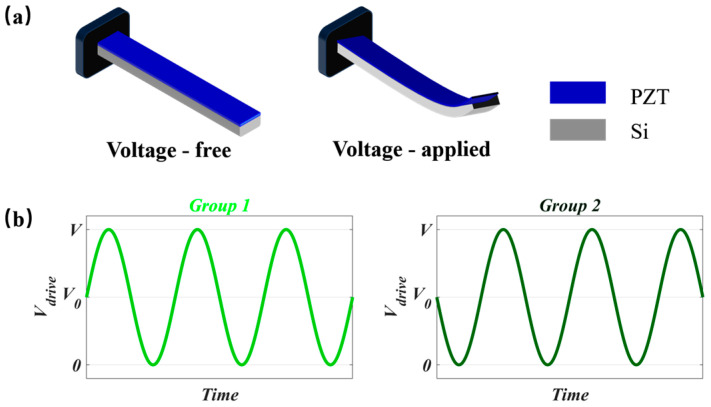
Piezoelectric drive: (**a**) principle of piezoelectric cantilever drive; (**b**) voltage signals with a 180° phase difference for two sets of drives.

The operating principle of the piezoelectric actuator is illustrated in Figure 5. The actuation structure consists of an Si cantilever beam with a patterned PZT deposited on its surface. The cantilever has a length of *L_pzt_*, with the thicknesses of the PZT and Si layers denoted as *t_pzt_* and *t_si_*, respectively. When a vertical (thickness-direction) electric field *E*_3_ is applied to the PZT, an in-plane strain *ε*_1_ is induced according to the inverse piezoelectric effect, described by

(5) $$\varepsilon_{1}=d_{31}E_{3},$$

where *d*_31_ is the transverse piezoelectric coefficient. The subscript “3” indicates the direction of the applied electric field, and “1” represents the direction of the induced strain (along the beam length). When the piezoelectric layer is subjected to strain, the mismatch in mechanical properties between the two layers causes the structure to bend, resulting in tip displacement *δ_st_*, as shown in Figure 5a. The tip displacement can be expressed as

(6) $$\delta_{\mathrm{st}}=\frac{3d_{31}s_{\mathrm{si}}s_{p}t_{\mathrm{si}}\left(t_{\mathrm{si}}+t_{p}\right)L^{2}V}{s_{\mathrm{si}}\left(2t_{p}^{4}+4s_{\mathrm{si}}s_{p}t_{\mathrm{si}}t_{p}^{3}+6s_{\mathrm{si}}s_{p}t_{\mathrm{si}}^{2}t_{p}^{2}+4s_{\mathrm{si}}s_{p}t_{\mathrm{si}}^{3}t_{p}+s_{p}^{2}t_{\mathrm{si}}^{4}\right)},$$

where *s_si_* and *s_p_* represent the mechanical compliance of the Si cantilever and the PZT film.

To achieve rotational actuation of the mirror about the *Y*-axis, as depicted in Figure 3, a uniform bias voltage is applied to Actuator Group 1. The PZT layer induces an upward bending displacement, which, through force transmission, generates a mechanical torque on the mirror that is anchored at both ends. Furthermore, by introducing a 180-degree phase delay between Actuator Groups 1 and 2 via external electronic control, selective control over the direction and amplitude of bending can be realized.

**Figure 6:**
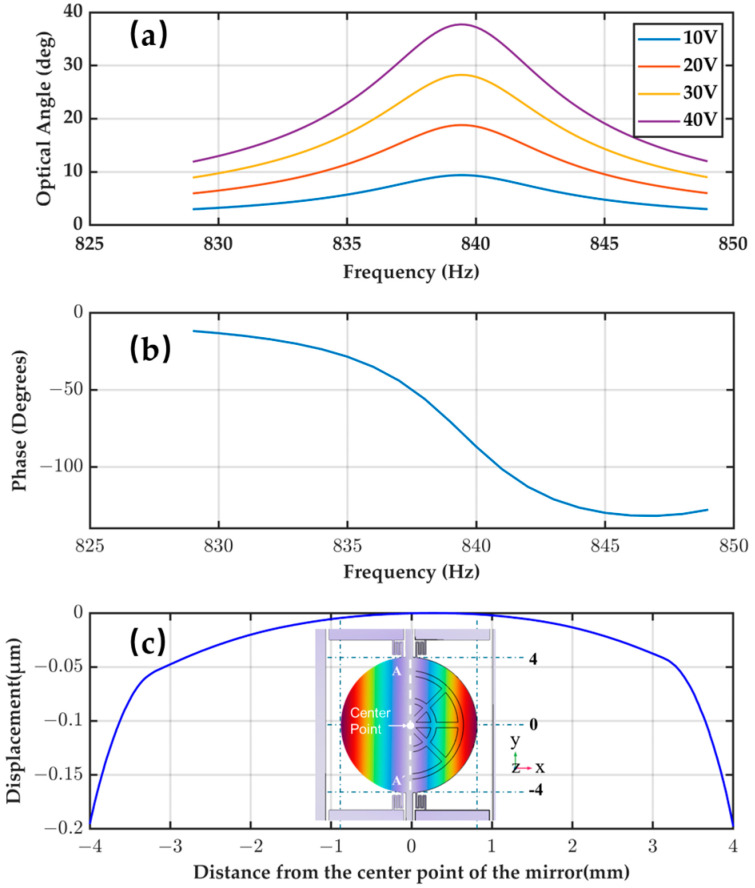
Piezoelectric fast-axis simulation: (**a**) frequency response curve, (**b**) phase analysis, (**c**) mirror axis deformation.

Based on the proposed design, FEA was conducted to analyze the angular response of the mirror under varying voltage biases. As shown in Figure 6a, the optical angle of the mirror increases with the driving voltage. This behavior is consistent with theoretical expectations: a larger electric field induces stronger piezoelectric strain, resulting in greater mechanical displacement.

Figure 6 shows the frequency response characteristics under electrical excitation, including the optical angle as a function of the drive frequency and the corresponding phase shift. As shown in Figure 6a, the angle exhibits resonant behavior, with a peak amplitude of approximately 39° occurring at a resonant frequency of around 838 Hz. The figure below shows the corresponding phase shift between the input drive signal and the optical angle. As the drive frequency increases from 830 Hz to 845 Hz, the phase response changes from approximately −15° to −145°. It is worth noting that a rapid phase drop occurs near the resonance frequency, where the phase shift is approximately −90°, consistent with the typical −90° phase shift observed in second-order resonant systems.

When studying fast-axis torsion, it is necessary to investigate whether there is parasitic motion. To this end, the displacement distribution of the micromirror surface along the *Y*-axis was calculated, with the results shown in Figure 6c. Along the *Y*-axis direction, a relative displacement of approximately 0.2 µm can be observed between the outer edge and central region of the mirror. This displacement primarily stems from structural deformation of the mirror plate rather than axis coupling effects. Therefore, it can be concluded that the proposed piezoelectric drive scheme is an effective strategy for mitigating the common issue in EM mirror designs.

A detailed comparison of this behavior with that of electromagnetic actuation is presented and discussed in Section 4, highlighting the improvements in scan fidelity achieved by the hybrid design.

#### 2.2.2. EM Drive

Vertical scanning of the mirror is achieved using an electromagnetic (EM) drive. A pair of permanent magnets is arranged along the Y direction of the structure to establish a uniform magnetic field, rather than an inclined one. This drive scheme is selected for the slow axis because piezoelectric actuators typically exhibit limited displacement and low efficiency under quasi-static or low-frequency conditions, whereas electromagnetic drives provide a more linear response and greater travel under similar conditions.

The slow-axis EM coil consists of 14 turns of gold wire, each 7 µm thick and 50 µm wide, wound in a planar spiral with 10 µm spacing between adjacent turns. The inner edges of the coil measure 10 mm and 15.5 mm for the short and long sides, respectively, while the outer edges reach 11.8 mm and 17.2 mm, resulting in a gradually increasing perimeter across turns. The total coil resistance is approximately 52 Ω, determined from the gold resistivity.

## 3. Device Fabrication

**Figure 7:**
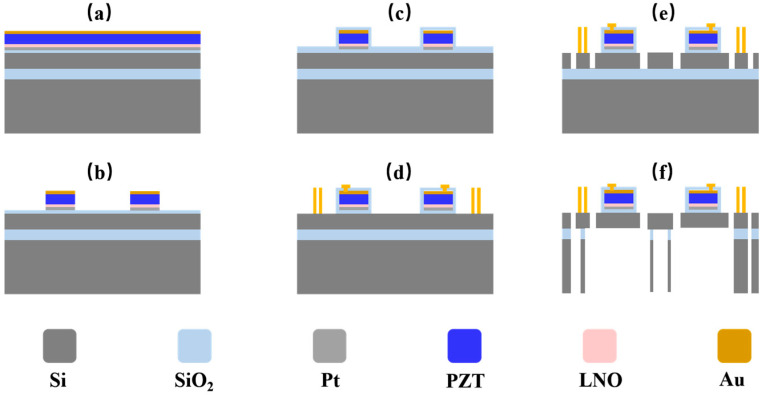
Manufacturing process flow: (**a**) preparation of the bottom electrode, seed layer LNO, piezoelectric layer PZT, and top electrode; (**b**) patterning of PZT and electrodes; (**c**) deposition of PECVD SiO_2_ on the top Au layer; (**d**) electroplating of coils; (**e**) DRIE etching of the device layer; (**f**) patterning and release of the handle layer and BOX.

Based on the prior design and FEM of the mirror structure, the starting substrate was a silicon-on-insulator (SOI) wafer, comprising a 60 μm device layer, a 1 μm buried oxide layer (BOX), and a 450 μm handle layer. The fabrication process is shown in Figure 7: (a) First, a 20 nm Ti and a 150 nm Pt bottom electrode and a 150 nm LNO were deposited on the device layer. Subsequently, a 2 μm PZT film was deposited on the top using magnetron sputtering [22]. The top electrode was formed by sequentially sputtering 40 nm of Cr and 150 nm of Au. (b) The top and bottom electrodes were patterned using ion beam etching (IBE), while the PZT layer was wet etched at room temperature using an HBF_4_ solution [23]. (c) A 1.5 μm SiO_2_ passivation layer was deposited over the entire wafer surface via plasma-enhanced chemical vapor deposition (PECVD) to prevent electrical shorts and protect the PZT layer. (d) Through reactive ion etching (RIE), through-holes were opened in the passivation layer, followed by electroplating to form interconnect lines and the slow-axis drive coils. (e) Through deep reactive ion etching (DRIE), front-side microstructures (including mirror panels, torsion beams, and drive frames) were etched in the 60 μm device layer down to the BOX layer. (f) DRIE etching was then performed on the back side of the wafer to form release cavities. The etching process automatically stopped at the BOX layer, which was subsequently removed via RIE to release the suspended structure.

**Figure 8:**
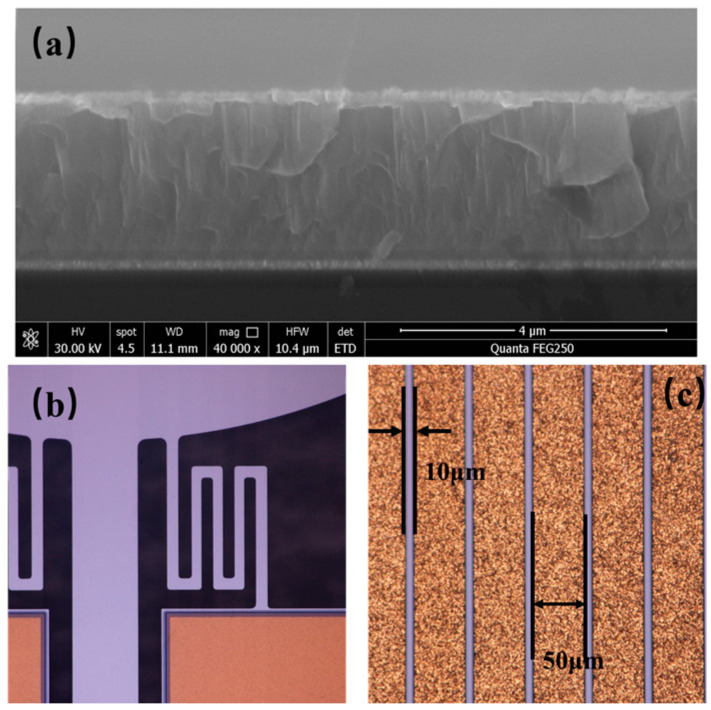
(**a**) PZT thin film cross-section, (**b**) S-type spring, (**c**) electroplated coil.

The completed mirror device exhibits precise structural definition and uniform layer integration. Scanning electron microscope (SEM) images of the prepared device are shown in Figure 8.

## 4. Discussion

### 4.1. Experimental Setup

**Figure 9:**
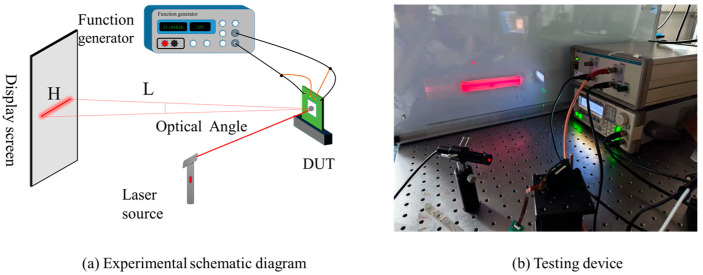
Experimental setup of the optical angle measurement system, including signal generator, amplifier, MEMS mirror, laser source (650 nm, 5 mW), and projection screen.

An optical measurement system was used to quantitatively evaluate the scanning distortion caused by slow-axis coupling during fast-axis excitation, as shown in Figure 9. The experimental platform consisted of a signal generator, a voltage amplifier, the MEMS mirror, a laser source (650 nm, 5 mW), and a projection screen for image acquisition. A voltage matching the fast-axis resonant frequency was generated, then applied to the driving pad of the mirror, inducing resonant motion along the fast axis.

The laser beam was directed onto the reflective surface of the mirror. As the mirror oscillated, the reflected beam traced a dynamic scan line in space, which was projected onto the screen. The optical angle can be calculated using the following formula:

(7) $$\theta_{\mathrm{opt}}=2\arctan(H/2L),$$

where *H* is the offset distance of the laser spot on the screen, and *L* = 40 cm is the vertical distance from the screen to the micromirror.

### 4.2. Scan Line Test

A comparative analysis was performed to evaluate the scan-line behavior of an electromagnetic mirror and a hybrid drive. The investigation focused on quantifying the displacement of the slow axis under conditions where only the fast axis was actively excited.

**Figure 10:**
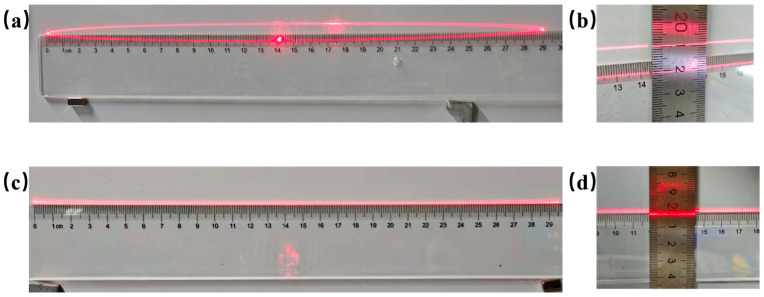
Fast-axis drive-scan trajectory: (**a**) 40° electromagnetic coupling scan line; (**b**) coupling scan cline vertical distance; (**c**) 40° piezoelectric drive scan line; (**d**) PE scan line vertical distance.

In the electromagnetic mirror, the application of a sine current to the fast axis resulted in a distinctly elliptical scanning pattern, as illustrated in Figure 10a. An optical deflection of 40° on the fast axis, with a resonant frequency of 726.9 Hz—corresponding to a 29 cm scan length on the projection screen—was accompanied by parasitic motion of the slow axis, leading to vertical beam spreading of approximately 1 cm, corresponding to an unintended optical angle of approximately 1.43°, as shown in Figure 10b.

By contrast, in the hybrid-driven micromirror, the fast axis was isolated using piezoelectric actuation. Under identical conditions, the laser trace appeared as a clean and narrow straight line, with no observable vertical spread, as presented in Figure 10c. In order to accurately quantify the vertical coupling behavior of the piezoelectric fast axis, the projection screen was moved to a distance of approximately 3 m from the micromirror, with geometric magnification used to expand the minute vertical displacement of the scan line to a resolvable scale. A CCD camera was aligned to capture the laser trajectory at three critical positions: the left edge, center, and right edge of the scan line. Subsequently, high-resolution images of these trajectories were processed to measure the vertical spread of the laser spot.

**Figure 11:**
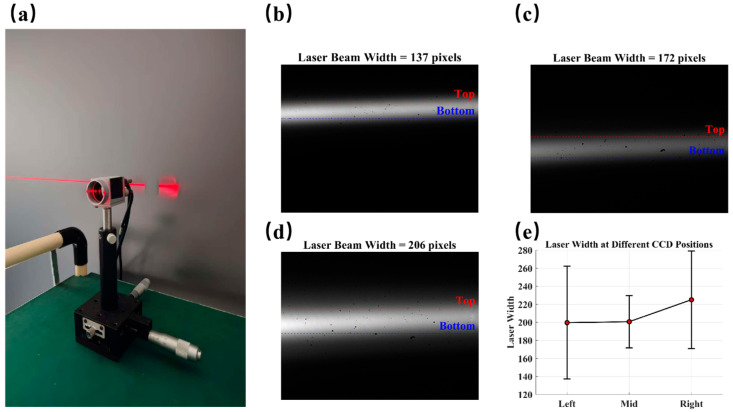
(**a**) CCD image capture settings; (**b**–**d**) RAW CCD images of the left, center, and right sides of the scan line, respectively; (**e**) average laser spot width at the three positions.

As shown in Figure 11e, the spot widths at all three positions are within the range of 137–270 pixels. This indicates that no significant trend of widening in the middle is observed, and the vertical diffusion of the laser point is still within a very small range.

The results show that, compared with the 1.43° parasitic deflection of traditional electromagnetic microscopes, the designed hybrid drive can effectively maintain the slow axis stationary during resonance fast-axis operation. This indicates that the slow axis remains effectively stationary, proving that mechanical crosstalk is significantly suppressed due to the decoupled drive mechanism.

**Figure 12:**
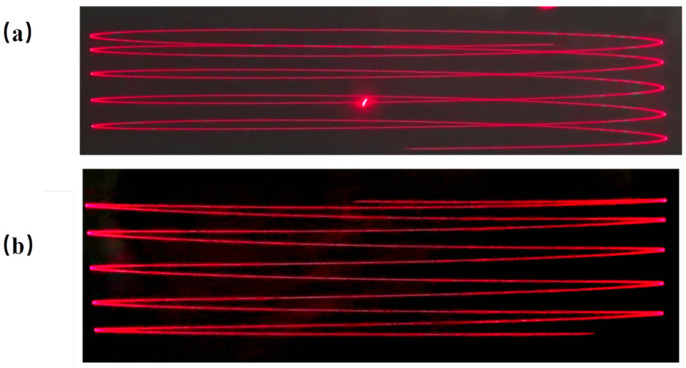
Raster scanning trajectory: (**a**) EM drive; (**b**) PE drive.

Figure 12 illustrates the schematic diagram of raster scanning achieved through a dual-axis drive. In Figure 12a, when using a single coil, the scanning trajectory exhibits a distinct overlapping reciprocating pattern. This indicates significant mechanical crosstalk between the axes. In contrast, Figure 12b shows the raster trajectory produced by the hybrid-driven mirror under the same driving conditions. It forms a clean rectangular grid with uniform line spacing, making this design suitable for optical scanning applications such as LiDAR.

Table 2 compares the proposed design with representative MEMS micromirrors reported in recent literature. The hybrid device achieves a favorable trade-off between large scan angle, high dynamic response, and mechanical stability.

### 4.3. Micromirror Characterization

**Figure 13:**
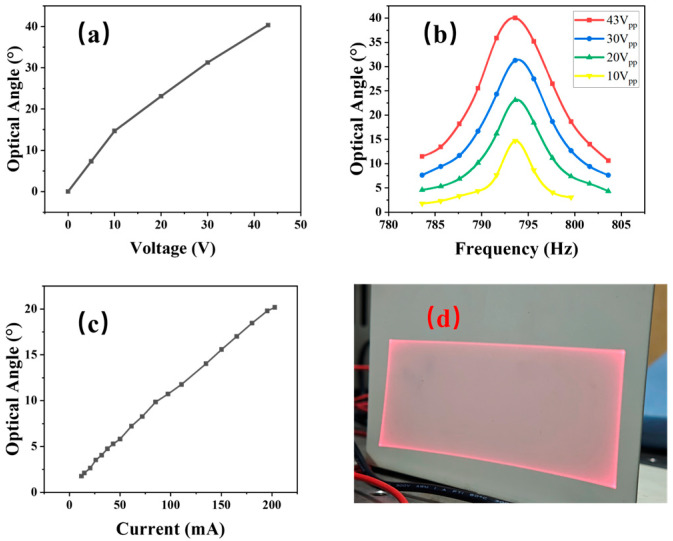
Hybrid drive test: (**a**) fast-axis voltage–angle curve; (**b**) fast-axis frequency–response curve; (**c**) slow-axis voltage–angle curve; (**d**) scan diagram.

As shown in Figure 13, the optical deflection angle of the fast axis increases linearly with the amplitude of the applied sinusoidal voltage. Within the test voltage *V_pp_* = 43 V, the mirror exhibits a maximum optical scanning angle of 40°, with no noticeable nonlinear behavior observed. Figure 13b shows the frequency response of the fast axis. A distinct resonance peak is observed at the design frequency (792 Hz range). The quality factor (Q) of the resonance, 110, ensures high angular deflection even with low signal excitation. Meanwhile, the slow axis undergoes quasi-static driving, with a linear relationship between voltage and angle, as shown in Figure 13c. At a driving frequency of 20 Hz, an optical angle of approximately 20° can be achieved.

When the fast axis is excited at its resonant frequency and the slow axis is simultaneously driven at 20 Hz, raster scanning is realized. As illustrated in Figure 13d, the proposed hybrid-actuated micromirror produces a well-defined rectangular scanning pattern.

## 5. Conclusions

This work presents a novel hybrid-actuated MEMS micromirror that integrates PZT and EM actuation to overcome mechanical crosstalk in conventional single-coil EM designs. By decoupling the drive mechanisms—utilizing PZT actuators with an S-shaped spring for resonant fast-axis scanning (792 Hz, Q ≈ 110, 40° optical angle) and EM coils for quasi-static slow-axis control (20° at 20 Hz)—the design achieves high-fidelity 2D scanning with minimal crosstalk interference. Finite element simulations and experimental validation confirm near-zero parasitic slow-axis deflection during fast-axis operation, reducing optical deviation from 1.43° in traditional EM mirrors to undetectable levels. Fabricated via an SOI-based process with integrated PZT thin films and electroplated coils, the device enables distortion-free 40° × 20° raster scans with uniform line spacing. This architecture provides a scalable solution for precision applications, including LiDAR and structured light projection.

The quasi-static driving capability of piezoelectric actuators remains relatively limited, which currently necessitates the use of electromagnetic actuation for slow-axis control. In addition, the hybrid drive introduces extra complexity in fabrication. Nevertheless, these drawbacks are likely to be alleviated as piezoelectric materials become more cost-effective and their stability and reliability continue to improve. Furthermore, compared with electromagnetic actuators that often require ion implantation for closed-loop feedback, piezoelectric devices inherently provide self-sensing capability, offering opportunities for simplified control schemes and reduced system cost. Taken together, the hybrid actuation strategy holds promise to achieve a more favorable balance between performance, reliability, and cost as materials and fabrication technologies mature, providing a practical pathway toward robust, large-aperture MEMS micromirrors for next-generation optical systems.

## Figures and Tables

**Table 1:** Micro mirror design parameters.

Design Parameter		Units
Mirror diameter	8	mm
Fast-axis width	400	μm
Fast-axis length	3500	μm
Piezoelectric width	4.3	mm
Piezoelectric length	2.4	mm
e31	15	C/m^2^
Slow-axis width	145	μm
Slow-axis length	1500	μm

**Table 2:** Comparison of this paper with different references.

References	Gu-Stoppel [16]	Liu [17]	Park [18]	Park [19]	Fang [20]	This study
Mirror diameter	1 mm	3 mm	3 mm	3 mm	7.2 mm	8 mm
Scan method	Lissajous	Lissajous	Raster	Raster	Raster	Raster
Principle	PE	PE	EM	EM	EM	PE
Drive signal	4 V	30 V	353 mA	188 mA	84.9 mA	43 V
Frequency	27 kHz	2061 Hz	902 Hz	1635 Hz	712 Hz	792 Hz
Angle	3.4°	32.8°	22°	50.6°	35.2°	40°
Decoupling effect	No coupling observed at 3.4°	No coupling observed at 32.8°	Reduced to 0.97%	Reduced to 0.45%	No coupling observed at 20°	No coupling observed at 40°

## Data Availability

The original contributions presented in the study are included in the article; further inquiries can be directed to the corresponding authors.
